# DiMER: a modular framework for replacing uninformative annotations in protein datasets

**DOI:** 10.1128/mra.00797-26

**Published:** 2026-08-31

**Authors:** Ellis L. Torrance, Kelly P. Williams, Joseph S. Schoeniger, Catherine M. Mageeney

**Affiliations:** 1 Systems Biology Department, Sandia National Laboratories 111651, Livermore, California, USA; 2 Biological, Radiation, and Signature Science, Technology, & Engineering Center, Sandia National Laboratories 111651, Livermore, California, USA; 3 Biotechnology & Bioengineering Department, Sandia National Laboratories 111651, Livermore, California, USA; University of Michigan, Ann Arbor, Michigan, USA

**Keywords:** annotation, microbes, phage, mobile elements

## Abstract

DiMER (https://github.com/sandialabs/DiMER) is a modular Python framework using user-provided BLAST and HMM databases to assign annotations while avoiding placeholders (e.g., hypothetical protein). DiMER was applied to a curated list of gold standard mobile genetic elements and increased functional annotations by 24% (49 to 73%).

## ANNOUNCEMENT

Accurate and informative functional annotation remains a central challenge in microbial genomics, paramount to understanding a microbe’s biological role in a community, identification of novel synthetic biology tools, and enhanced metabolomic and interaction modeling (1). This challenge is particularly difficult for mobile genetic elements (MGEs), such as prophages and integrative conjugative elements, since they contain large amounts of “dark matter” (1). Homology-based annotation is standard practice, but many microbial sequences still retain large fractions of coding sequences labeled with uninformative annotations (termed placeholders; e.g., “hypothetical protein” or “uncharacterized protein”) (1). These labels persist, even when more informative homolog-derived descriptions exist, because many annotation workflows select only the single best-scoring hit per query and do not explicitly discriminate against placeholder terms. Consequently, downstream comparative analyses and functional interpretation of MGE gene inventories are hindered, and placeholder terms are propagated in derived databases and training sets.

**Fig 1:**
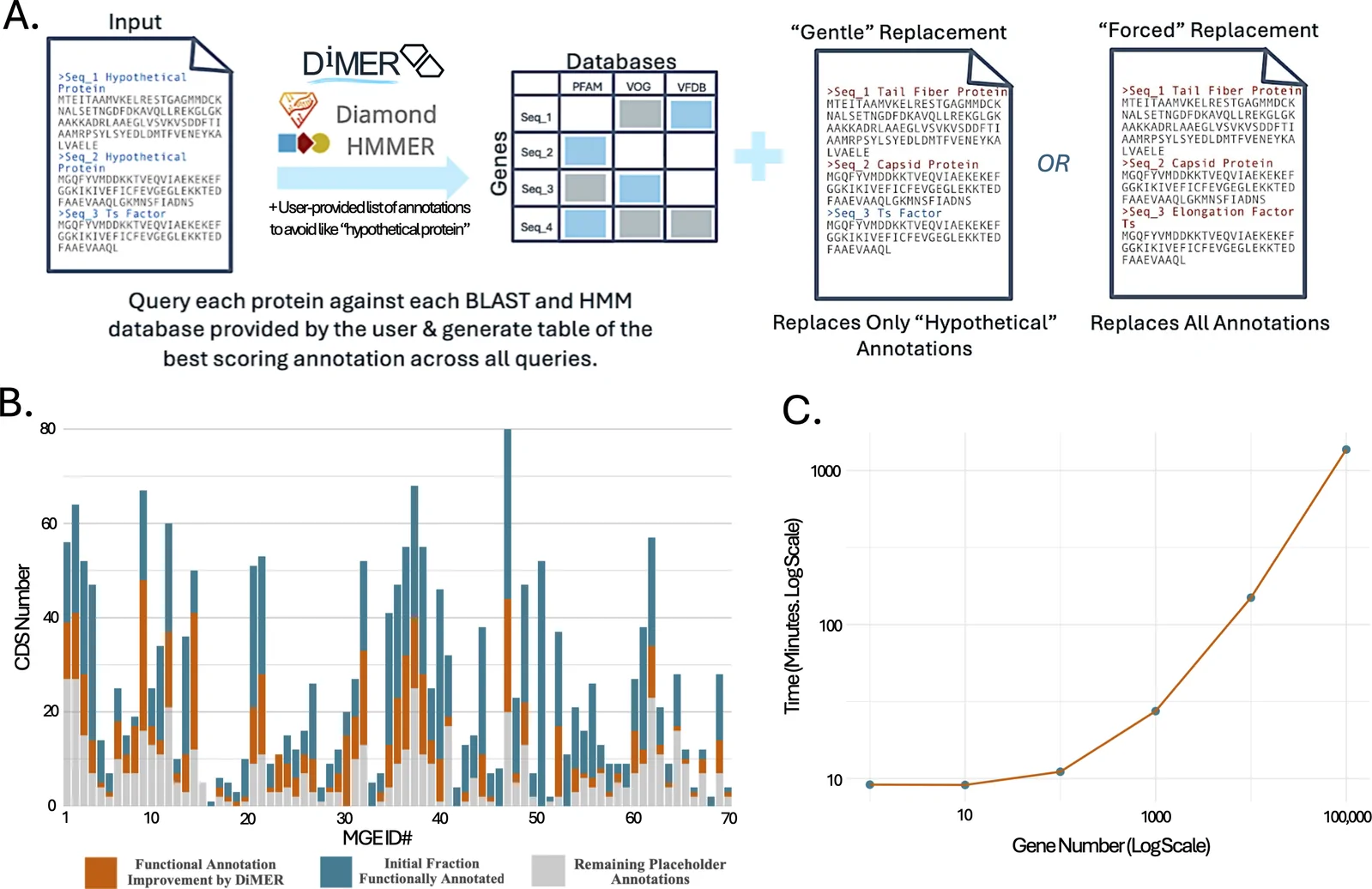
Overview and performance metrics of DiMER. (**A**) DiMER queries user-provided databases to annotate proteins while selectively avoiding annotations deemed as placeholders whenever possible. (**B**) Stacked bar plot demonstrating replacement of placeholder annotations in bacterial MGEs (*n* = 79) using DiMER. (**C**) Plot demonstrating DiMER runtime in minutes across a randomly generated data set of differing numbers of genes in log scale.

We developed DiMER, a modular Python framework intended to quickly reduce placeholder annotation retention by explicitly incorporating user knowledge of their genomic system to identify uninformative annotations. DiMER takes a protein FASTA file (with or without existing annotations) and queries user-specified databases in HMMER or DIAMOND format (2, 3). For each query protein, DiMER records all best matches to the queried databases in a single table and selects a single final annotation using e-value ranking under a user-defined threshold (default: 1e−10) (Fig. 1A). During this selection, DiMER preferentially avoids any user-defined placeholder annotations (listed in placeholders.txt) and retains the best available hit.

DiMER can run in “gentle” mode where it will replace only the initial gene annotations that were identified as placeholders (via placeholders.txt) or in “force” mode, which replaces all annotations based on e-value across the queried databases while still preferentially avoiding placeholders (via placeholders.txt) (Fig. 1A). DiMER supports iterative refinement where users can update the uninformative annotation list based on annotations encountered in the DiMER output (e.g., “DUF#” or “ORF#”). DiMER can be rerun to improve annotation without repeating expensive database searches when prior results are present. DiMER was built using Python 3 and requires HMMER v3.1b2 (2), Diamond v2.1.10 (3), and the Python library Pandas to run. Installation and running are straightforward, and instructions are available at https://github.com/sandialabs/DiMER.

As a demonstration, DiMER was evaluated on experimentally validated MGEs (*n* = 79) (4) downloaded from the National Center for Biotechnology Information (NCBI) that contained 1,998 predicted ORFs. Of these, 1,021 (51%) were labeled with placeholder terms by NCBI/PGAP at download. We compiled 15 MGE-relevant databases (three DIAMOND-formatted protein databases and 12 HMM profile databases [5–20]) and ran DiMER in gentle mode, replacing 483 placeholder annotations (47% of the placeholder fraction) and increasing the functionally annotated proportion from 49 to 73% (Fig. 1B). Furthermore, with these databases, DiMER required only ~10 min to run on genetic data sets with <1,000 genes (Fig. 1C). These results demonstrate the utility of DiMER to improve MGE annotation. Furthermore, this software can be easily implemented into existing annotation workflows for any organism of interest and reduces time searching across multiple databases for the most informative and functionally accurate annotations.

## Data Availability

The dataset of MGE analyzed in this publication was taken from Langille et al. (2008) (4) and downloaded from NCBI by accession number (listed at: https://doi.org/10.5281/zenodo.15724972). The software DiMER and the associated databases used in this analysis are available at github.com/sandialabs/DiMER under the CC0-1.0 license. A copy with an associate DOI is available at https://doi.org/10.5281/zenodo.21893019.

## EDITOR'S NOTE

**Ed Note**: This article was handled by Pat Schloss, who acted as a guest editor in consultation with the editor in chief, Irene Newton. Dr. Schloss's role as the guest editor of this article is outside of his duties as the chair of the Journals Committee.
